# Two Interesting Cases of Capped Pulps

**Published:** 1897-06

**Authors:** R. E. Sparks

**Affiliations:** Kingston, Ont.


ARTICLE II.

### TWO INTERESTING CASES OF CAPPED PULPS.

BY R. E. SPARKS, M. D., D. D. S., KINGSTON, ONT.

My experience of capping exposed pulps has not been of the most encouraging character. After practicing it for twenty years, in all cases where there seemed to be any probability of success, I am led to wonder who extracts or treats the failures of those who report almost universal success.

I think I get a fair share of cases; and, as I said before, I cap all cases where there promises to be a probability of success. I have tried all methods that I have read of, or heard advocated. While I recognize the advantage of a tooth having a living pulp over one from which the pulp has been removed, I must confess that, from my experience, I feel much more confident of success when I have made an application to devitalize an exposed pulp than when I have capped it.

Two cases came under my notice recently, which illustrates this :

Case 1. Miss M., aged about twelve, came to have her tooth filled, on February 27th, 1890. Among other cavities was one on the anterior surface of the left lateral incisor. It was only an ordinary sized cavity, and had never given any trouble. When I removed the decay near the cutting edge, I was surprised to find I had made a slight exposure of the pulp. I capped it. It gave no trouble. On July 8th, 1891 I refilled it with ceme t. On March 9th, 1894, I refilled it with gold, everything being, apparently, in a satisfactory condition. Until about the new year, 1897, I would have reported this an unqualified success. At about this date the young lady came to my office complaining of severe pain in the tooth. I found the gum swollen above it; the tooth somewhat loose. The color had remained remarkably good. The characteristic opacity was present, however, upon close inspection. This, with the other symptoms, convinced me that my grand success, as in many other cases, was an utter failure. Upon opening into the pulp chamber, pus boiled out profusely. After thoroughly cleansing and a few daily antiseptic treatments, the root was packed with cotton, saturated with eucalyptol and closed up until the 16th, when it was refilled permanently, and, so far, is perfectly comfortable.

Case 2. Dr. R., aged about 65, came complaining that between his first and second molars, on the left side of the lower jaw, food would wedge, and was difficult to remove Upon examination I found the gum receded considerably from his teeth, making large spaces between them. In the space referred to, I found a large cavity on the posterior surface of the first molar, about the neck, opening out well to the lingual side. I removed the decay from this opening. At one point it was very sensitive. I suspected that the decay had reached the pulp, but as the tooth had caused him no pain before, I filled the cavity with cement. About two months afterwards he reported that from the time the tooth was filled it was sensitive to thermal changes; that it had become so much so that he had no comfort with his meals. It pained when he went out doors and again when he came in. I was convinced that the pulp must be devitalized. I opened through the grinding surface; a slight shock was caused when the drill opened into the pulp chamber. The main body of the pulp was removed without pain, and the opening enlarged. I found a little nerve matter not entirely dead in the root canals. I dropped a drop of the saturated solution of cocaine in carbolic acid into the pulp chamber and after a little patient working with a broach was enabled to remove the balance of the nerves. After drying the canals with cotton on broaches, followed by hot air, I swabbed them out with eucalyptol, and filled with chloropercha and gutta percha points, at the same filling. This was some months since, and the doctor reports having had no trouble whatever since the operation.—*Dominion Dental Journal.*

